# Phenology and Spatial Genetic Structure of *Anadenanthera colubrina* (Vell.), a Resilient Species Amid Territorial Transformation in an Urban Deciduous Forest of Southeastern Brazil

**DOI:** 10.3390/genes16040388

**Published:** 2025-03-28

**Authors:** Ana Lilia Alzate-Marin, Paulo Augusto Bomfim Rodrigues, Fabio Alberto Alzate-Martinez, Gabriel Pinheiro Machado, Carlos Alberto Martinez, Fernando Bonifácio-Anacleto

**Affiliations:** 1 Plant Genetics Laboratory, Department of Genetics, Faculty of Medicine of Ribeirão Preto (FMRP-USP/RP), University of São Paulo, Ribeirão Preto 14049-900, SP, Brazil; paulobomfim21@hotmail.com (P.A.B.R.); gabrielpm_4@hotmail.com (G.P.M.); 2 Graduate Program, Department of Genetics, Faculty of Medicine of Ribeirão Preto (FMRP-USP/RP), University of São Paulo, Av. Bandeirantes 3900, Ribeirão Preto 14049-900, SP, Brazil; 3 Nawi Spatial Design and Research, Ribeirão Preto 14040-160, SP, Brazil; nawistudio.contact@gmail.com; 4 Department of Biology, Ribeirão Preto School of Philosophy, Science and Literature (FFCLRP), University of São Paulo, Av Bandeirantes 3900, Ribeirão Preto 14040-901, SP, Brazil; carlosamh@ffclrp.usp.br

**Keywords:** *Anadenanthera*, forest conservation, genetic diversity, molecular markers, seasonally dry tropical forests—SDTF, pollinator network, landscape genetics

## Abstract

Background/Objectives: *Anadenanthera colubrina* (popularly known as angico; in this study: Acol) is a bee-pollinated tree with gravity-dispersed seeds that occurs in dry tropical forests (SDTF), one of the most fragmented tropical ecosystems. In this study, we analyzed the resilience of 30 Acol Forest fragments of Ribeirão Preto, São Paulo, Brazil, and the flow of pollinators among these fragments based on the flight ranges of *Apis mellifera* (6 km) and *Trigona spinipes* (8 km). Additionally, we investigated genetic diversity, spatial genetic structure (SGS), and phenology across generations of one Acol population (AcolPM), located in the urban fragment M103 in the “Parque Municipal Morro de São Bento” (a municipal park in Ribeirão Preto). Methods: We mapped Acol fragments using geospatial data, with relief and slope analysis derived from digital terrain modeling. We created a flow diagram based on the pollinator’s flight ranges and calculated betweenness centrality. We amplified DNA from AcolPM individuals using 14 SSR molecular markers. Results: Notably, 17 of the 30 fragments occurred on slopes > 12%, terrain unsuitable for agriculture or construction, indicating that the presence of *A. colubrina* may serve as an indicator of territorial transformations. The AcolPM population (Fragment M103) emerged as a key node among the *angicais*, connected by the native pollinator *T. spinipes*, being fundamental for regional gene flow. In this focal population, we observed a slight but significant inbreeding (*Fis*, *Fit*, *p* < 0.01) and an SGS up to ~17 m. Genetic diversity was intermediate (He ≈ 0.62), and PCoA, Fst, and AMOVA values suggest low generational isolation, with most genetic variation within generations. This highlights AcolPM as a promising source for seed collection for reforestation. Phenological observations showed that fructification occurs between September and October, at the beginning of the rainy season. Conclusions: We concluded that Acol resilience is linked to the species’ mixed-mating system and pollinator dynamics-driven connectivity, allowing for the maintenance of genetic diversity in fragmented landscapes, as well as its natural tendency to form dense *angicais* clusters in non-arable slopes. We reaffirmed *A. colubrina* as a valuable species for restoration and urban climate resilience, providing cooling shade to humans and wildlife alike while offering refuge and food for local insects and birds in a warming landscape.

## 1. Introduction

Habitat fragmentation, caused by intensive agriculture or urban expansion, affects the size and spatial separation of plant populations, leading to genetic diversity loss and increased genetic differences between populations due to higher random genetic drift, elevated inbreeding, and reduced gene flow [1]. Limited gene flow is the most prevalent cause of spatial genetic structure (SGS). SGS refers to the non-random distribution of genotypes in a two-dimensional space that results in the formation of local neighborhoods of genetically related individuals [2]. Physical distance and the dispersal capacity of pollen and seed agents influence the extent of gene flow among forest fragments. In outcrossing species, restricted seed or pollen movement can lead to inbreeding due to the proximity of related individuals or mating among relatives, thus affecting SGS [3].

One of the most fragmented and vulnerable tropical terrestrial ecosystems is the Seasonally Dry Tropical Forest (SDTF), which usually includes individuals belonging to *Anadenanthera*, a tree genus endemic to Latin America and the Caribbean [4,5,6]. *Anadenanthera colubrina* (Vell.) Brenan, commonly known as angico, is a tropical heliophilous species from the Leguminosae-Mimosoideae family that tolerates sandy and shallow soils and light shading in the juvenile phase, reaching up to 35 m in height [7,8]. It has the greatest geographic range within its genus, occurring from southern Bolivia to northern Argentina at altitudes between 15 and 2000 m; in Brazil, it does not appear in the southern states. *A. colubrina* is a fast-growing species, maturing around the age of eight years [8], resulting in *A. colubrina* populations named *angicais* being composed of individuals from several generations, whose spatial distribution reflects a relatively recent gene flow [9].

*A. colubrina* aggregates flowers into axillary or terminal flower heads, has staminate flowers at the base, and has perfect flowers at the apex of the inflorescence, characterizing it as andromonoecious [10]. *A. colubrina* is naturally pollinated by the generalist tropical stingless bee *Trigona spinipes* (Fabricius, 1793) (Apidae: Meliponini) and the honey bee *Apis mellifera* (Linnaeus, 1758) [7], with geitonogamy being a common behavior for both species [11,12]. These bees can forage up to 30 m in height [13]. It has been documented that *T. spinipes* can fly between 840 m and 8 km [14,15], while *A. mellifera* has a foraging range from 45 m to 6 km, depending on the time of year and colony size (multiple authors cited by [16]). *T. spinipes* performs better in disturbed than in preserved habitats and could serve as a rescue pollinator, able to compensate for the decline of other less resilient pollinators in degraded habitats [15]. *A. colubrina* seeds primarily rely on autochorous dispersal, with barochory being the main mechanism [8].

The phenology of *A. colubrina* shows broad variation, generally occurring over several months in the second half of the year, depending on the location of occurrence in Brazil [5]. In this sense, its regional phenological characterization is crucial for its use in reforestation and seed collection and to monitor future changes over time that may impact its survival. In the state of São Paulo, Brazil, *A. colubrina* is listed among the species for reforestation [17], being used for reforesting parks and planting mixed forests aimed at restoring degraded areas, especially riparian forests, and as part of agroforestry systems.

Ribeirão Preto, located in the interior of São Paulo State, southeastern Brazil, is an important economic region covering 651 km^2^, with its soils used for the industrial production of ethanol from sugarcane. It is one of the most anthropogenically devastated forest landscapes in the state. Removing natural vegetation in the Ribeirão Preto region began more than 100 years ago during coffee exploitation [18,19]. Currently, due to urban expansion, the remaining natural vegetation is highly fragmented and exposed to continued degradation. Among the 102 remaining natural forest fragments in Ribeirão Preto, the M103 fragment [18], located in the “Parque Municipal Morro de São Bento” (a municipal park in Ribeirão Preto, São Paulo State, southeastern Brazil), acquired by the Ribeirão Preto municipality in 1907, stands out as one of the last patches of native vegetation in an urban area. M103 spans 9000 m^2^ and comprises primarily a deciduous forest on the hilltop, characterized by litholic and shallow soils, and 3 hectares of semi-deciduous forest on the hillside with deeper soils. Within the deciduous forest of the “Parque Municipal”, *A. colubrina* dominates as a key species, and this population has been designated as AcolPM.

Previous research by our group on two *A. colubrina* populations, a population located on the campus of the University of Sao Paulo (USP) at Ribeirão Preto city, São Paulo (AcolUSP), and a small population of *A. colubrina* from a rural area with recent deforestation near the district of Bonfim Paulista, SP (AcolBP) using SSR markers (simple sequence repeat), identified intermediate and similar diversity indices and inbreeding [20]. Mating rate studies in the AcolBP population showed that *A. colubrina* undergoes a mixed-mating system (tm Acol = 0.619), with a wide range (from 0.196 to 1) in the estimate of tm when analyzing 10 families, reflecting its plasticity, and an additionally observed 15% of mating among relatives (tm-ts) [6]. Later, ref. [21], using ISSR (inter simple sequence repeat) molecular markers, observed similar diversity levels (~h = 0.30) in the same two populations and in a sample of adult individuals from AcolPM. However, the UPGMA dendrogram showed that AcolPM individuals formed a distinct cluster despite the shorter physical distance (~6 km) between AcolPM and the other two populations. Also, AcolPM exhibited greater genetic distances (~5%) from AcolUSP and AcolBP, compared to the smaller genetic distance observed between AcolUSP and AcolBP (2.4%), which are separated by a physical distance of 14 km [21].

In this research, we aimed to analyze the resilience of *angicais* in the region amid forest fragmentation. We studied the causes of their widespread occurrence in forest fragments of Ribeirão Preto and analyzed the genetic diversity, spatial genetic structure, and phenology characteristics of the most anthropogenically altered fragment of *A. colubrina* (AcolPM). This study used 14 SSR molecular markers in three generations, integrating all this information to understand the resilience of the species in an extremely affected environment.

We hypothesized that the generations of this isolated population of *A. colubrina* exhibit genetic spatial distribution patterns due to its natural mating, barochoric seed dispersal system, typical species aggregation in clusters, and inbreeding from mating between relatives. These characteristics might be intertwined with the occurrence of *A. colubrina* on hilltops and difficult reliefs, marginal to land-use exploitation, as observed during field visits. However, its natural mixed-mating system and pollinators (which fly long distances), along with the occurrence of *A. colubrina* populations in the neighborhood, maintain gene flow connections with the nearest populations (about 8 km away) conserved on hilltops in the Ribeirão Preto landscape, making it a resilient species in isolated populations.

## 2. Materials and Methods

### 2.1. Analysis of Fragments with A. colubrina Populations in Ribeirão Preto, SP, Brazil

**Figure 1:**
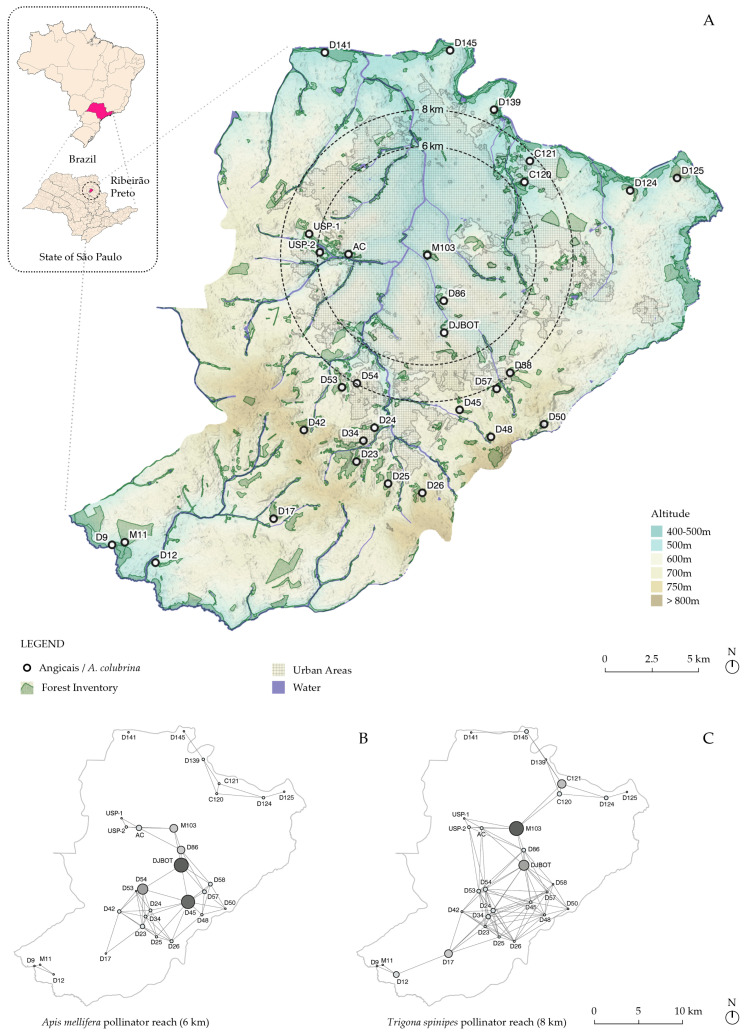
(**A**) Geographic localization of fragments with *Anadenanthera colubrina* (Vell.) occurrences in the Ribeirão Preto region, SP, Brazil (IBGE [25]). Except for USP1, USP2, and AC, all localizations are based on a previous characterization [18]. (**B**,**C**). Betweenness centrality analysis of bees’ flow connectivity within the pollination network considering the documented maximum flight ranges of *Apis mellifera* (6 km, (**B**)) and *Trigona spinipes* (8 km, (**C**)). Maps were created using QGIS 3 with field-collected and geospatial datasets [22,23,24,25,26].

We utilized information on the occurrence of *A. colubrina* from Kotchekoff-Henriques (2003) [18] and additional data gathered by our research group to compile a list of population fragments (Table S1). These fragments were mapped within their landscape context using geospatial datasets [22,23,24,25,26], and a relief and slope analysis was performed with a digital terrain model at 30 × 30 m resolution [27] (Figure 1, Table S1). Additionally, we created a flow diagram among the fragments based on the documented maximum flight ranges of *A. mellifera* (6 km) [16] and *T. spinipes* (8 km) [15], key pollinators of *A. colubrina*. To assess connectivity within the pollination network, we calculated betweenness centrality using Gephi 0.10 software (https://gephi.org). This metric identifies nodes that act as bridges by appearing on the shortest paths between other nodes, reflecting their influence on the overall flow within the network. Each node receives a quantitative score, with higher values indicating greater importance in promoting connectivity and facilitating gene flow.

The results are visually presented in a graph, where larger or more prominent nodes represent central hubs. Geospatial data were mapped and analyzed using QGIS 3, with all coordinates georeferenced under the SIRGAS 2000 23S system.

### 2.2. Identification and Sampling of A. colubrina Populations

**Figure 2:**
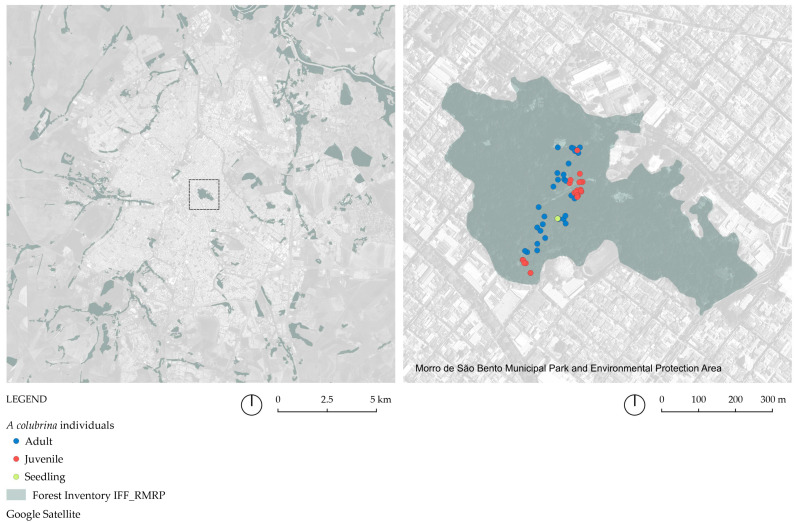
Geographic location of the sampled *A. colubrina* (Vell.) Brenan individuals from the AcolPM population, located in the M103 fragment, which is part of the Parque Municipal Morro de São Bento, Ribeirão Preto, SP. The maps were created in QGIS 3 using field-collected data and satellite imagery from Google Earth.

**Figure 3:**
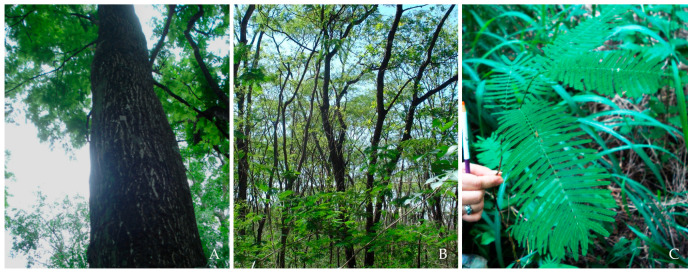
Adult (**A**), juvenile (**B**), and seedling (**C**) individuals of *A. colubrina* from the AcolPM population located in the M103 fragment, part of the “Parque Municipal Morro de São Bento”, Ribeirão Preto, SP, Brazil.

We randomly sampled leaves from 69 individuals of an *A. colubrina* population (referred to as AcolPM in this study) located within a deciduous forest fragment in an urban area of the “Parque Municipal Morro de São Bento”, Ribeirão Preto, São Paulo State, Southeastern Brazil (coordinates: 23 K 209123.25 mE, 7656134.68 mS, altitude: 560 m). This forest forms part of the larger M103 fragment, previously characterized by Kotchekoff-Henriques [18] (see Figure 2). Most of these trees are protected by a fence. We verified the species identification using the species-specific molecular marker ISSR UBC2_250 bp_ and the morphological characteristics, such as their brightly colored fruits, according to [21]. The 30 adult trees were reproductive individuals of significant height, dominating the canopy of the fragment (DBH_mean_ = 1.99 m); the 22 juvenile non-reproductive trees had well-developed canopies and trunks (DBH_mean_ = 0.73 m), and the 17 seedlings had a height between 0.5 and 1.20 m (Figure 3, Table S2). The individuals were georeferenced by GPS (eTrex Vista Cx, Garmin, Olathe, KS, USA) and identified as Acol (*A. colubrina*), with a specific numbering (Figure 2).

### 2.3. Phenological Observations

Phenological surveys of *A. colubrina* were conducted over 12 months from September 2014 to October 2015. Due to the height of the trees, which can reach up to 35 m, we selected 10 adults (DBH_mean_ = 170 cm) and 10 juveniles (DBH_mean_ = 55.81 cm) in the AcolPM population, which were visually accessible to facilitate inspections, located within an approximate radius of 70 m (Table S3). These trees were visited weekly. To further confirm the phenophase periods, the entire area was surveyed, including the marked individuals and the surrounding population, to ensure comprehensive coverage of the recorded characteristics.

Aiming the integration between meteorological data to explore potential correlations between climatic conditions and the timing and intensity of phenological events, we included temperature, relative humidity, and precipitation (http://www.ciiagro.org.br/mensal/cmensal (accessed on 15 February 2025)) [28] for the studied period.

### 2.4. DNA Extraction, Amplification, and Electrophoresis

We collected the leaves of the *A. colubrina* individuals and stored them at −20 °C until DNA extraction, which was performed using an adapted CTAB protocol for genomic DNA extraction according to Alzate-Marin et al. in 2009 [29] and quantified with a NanoDrop Spectrophotometer (ThermoScientific, Waltham, MA, USA). We amplified the DNA samples with 14 pairs of SSR primers (Acol 2, 5, 9, 10, 14, 11, 12, 13, 15, 16, 17, 18, 19, 20 [20,30]) in a final volume of 12 μL using the GoTaq^®^ Kit Promega (Promega, Madison, WI, USA), comprised of 5 μL of nuclease-free water, 5 μL of master mix [400 nM of each deoxynucleotide and 3.0 mM of MgCl2], 1 μL of each primer, and 2.0 ng/μL of genomic DNA (Feres et al., 2012 [30]). We performed the PCR amplification reactions in a Mastercycler^®^ pro-S Eppendorf thermocycler (Eppendorf, Hamburg, Germany) following these conditions: 1 cycle at 96 °C for 3 min, 30 cycles of denaturation at 94 °C for 30 s, annealing from 52 to 60 °C for 45 s (Table S4) followed by 72 °C for 1 min, and final extension at 72 °C for 7 min [20]. Amplicons (10 μL) were denatured and separated on 10% denaturing poly-acrylamide gels stained with silver nitrate [31]. Allele sizes were estimated by comparison with a 10-bp DNA ladder (Invitrogen, Carlsbad, CA, USA).

### 2.5. Statistical Analyses

We calculated genetic diversity parameters [average (Na) and effective (Ne) number of alleles per locus, observed (Ho) and expected (He) heterozygosity], fixation index (F), Nm (migrants per generation), AMOVA, and Nei’s unbiased measures of genetic distance using the GenALEx software version 6.51b2 [32]. Using the software FSTAT version 2.9.4 [33], we assessed F–statistics (Fis, Fit, and Fst among plants within populations according to [34]), and to verify whether the average estimates were statistically different from zero, the 95 and 99% confidence intervals were calculated using the bootstrap method (10,000 repetitions over loci).

We generated a distance matrix using the GenAlex 6.51b2 software, and from that, we constructed a scatterplot by Principal Coordinate Analysis (PCoA) and a genetic dissimilarity dendrogram based on the UPGMA algorithm [35] with the software MEGA version 11 [36]. We used the PAST software version 4.03 [37] to test the normal distribution of the data and the differences between the indexes among the groups with ANOVA.

We analyzed the spatial genetic distribution of genotypes (spatial genetic structure—SGS) for the adult and juvenile seedling generations, as well as for the entire population, using multilocus and multiallelic spatial autocorrelation analysis. This analysis, performed with the GenALEx 6.51b2 software [32,38], was based on genetic and geographic distances calculated from GPS coordinates. The software calculates an autocorrelation coefficient (*r*) to evaluate the relationship between genetic and geographic distances. The coefficient (*r*) quantifies the genetic similarity between individuals within distance classes, ranging from −1 (negative correlation) to 1 (positive correlation), with 0 indicating no correlation. The statistical significance of *r* was assessed using 1000 random permutations and bootstrapping.

## 3. Results

### 3.1. Mapping the Regional Fragments with A. colubrina Occurrence

**Figure 4:**
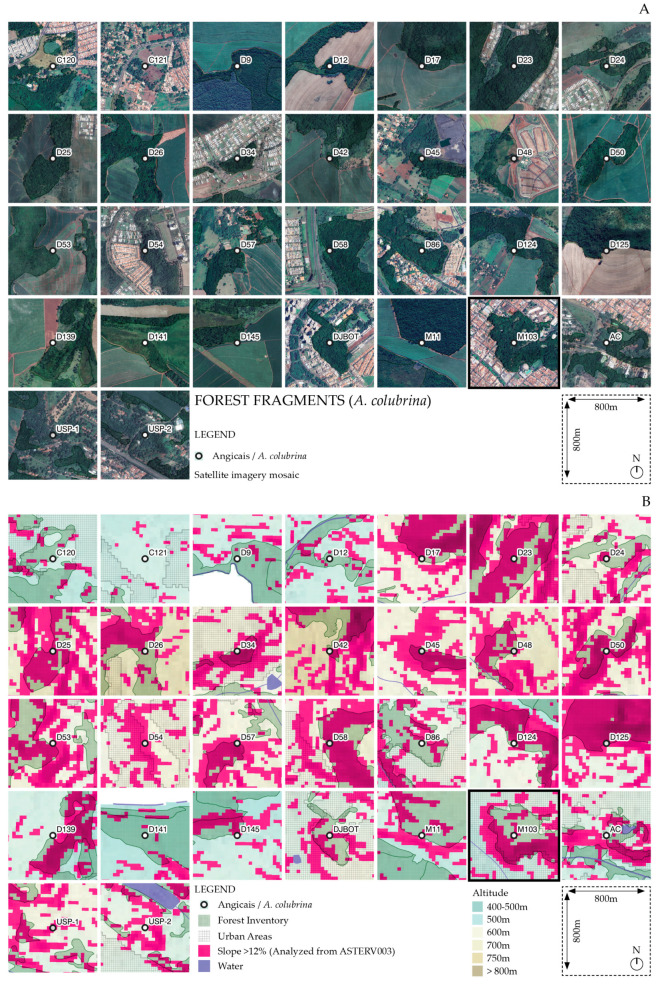
(**A**) Geographical localization of 30 fragments with high *A. colubrina* occurrence in the Ribeirão Preto, SP, Brazil region. Except for USP1, USP2, and AC, all localizations were according to previous characterization [18]. In (**B**), it is notable that the majority of these fragments, including M103, occurred on slopes greater than 12% (purple color), locations unsuitable to implement mechanized sugarcane plantation [39], indirectly resulting in their conservation. The maps were created in QGIS 3 using field-collected data, Google Satellite, and geospatial datasets [22,23,24,25,26].

The occurrence map of the fragments (Figure 1A and Figure 4A) shows that 15 out of the 30 fragments were located in urbanized landscapes, with three in the most densely populated region (M103, D86, and DJBot). Agricultural land use surrounded the other 15 (Figure 4A). The flow diagrams among the fragments, using the documented maximum flight ranges of the *A. colubrina* pollinators *A. mellifera* and *T. spinipes*, demonstrated that the 8 km network from the native pollinator encompassed all fragments, and M103 assumed a critical node for a hypothetical gene flow among them (Figure 1B,C). When examining the slope of the fragments, it was observed that 17 were located on slopes greater than 12% (Figure 4B), which comprise terrains unsuitable for mechanized sugarcane plantation according to São Paulo state legislation [39].

### 3.2. Phenological Events

The phenological observations of *A. colubrina* individuals from the AcolPM population that dominates the deciduous forest portion of the M103 fragment showed that, at the beginning of September 2014, the trees were leafless, with greater intensity in juveniles, since the rate and degree of rehydration varied strongly with the availability of water stored in tree trunks or in the subsoil [40]. In October, both adult and juvenile populations developed new leaves and large branches. However, in juveniles, leaf development continued until November, when the canopy closed.

**Figure 5:**
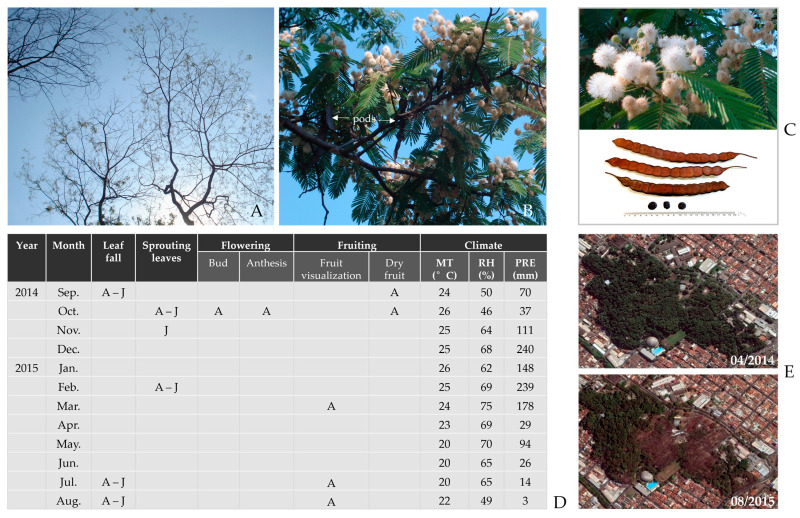
Initial sprouting and dry pod (**A**) and flowering (October 2014) (**B**) in adult individuals of *A. colubrina* from the AcolPM population in the M103 fragment. In (**B**), all stages of flower development are visible simultaneously, from the initial white phase to their aging, when they turn brown before wilting and falling. Mature fruits from the previous year’s flowering are also observed. In (**C**), details of buds, flowers, pods, and seeds are shown. (**D**) Phenology and climate data for the period (Mean Temperature [°C]—MT, Relative Humidity [%]—RH, Precipitation [mm]—PRE) are presented (http://www.ciiagro.org.br/mensal/cmensal, [28]). (**E**) Historical images from Google Earth depict the deciduous area of M103 fragment, Ribeirão Preto, SP, showing the canopy filled with leaves in April 2014 and completely defoliated in August 2015.

In the same period, October 2014, after the emergence of new leaves, intense flowering was observed only in adults (Figure 5), accompanied by abundant insect visitation in search of floral resources. During this time, several phenophases coincided, from flower bud development to flower opening (white when newly opened and brown when aged) until they fell (Figure 5B,C). This phenophase occurred under climate conditions of an average temperature of 26 °C (T), an average relative humidity of 46% (RH), and precipitation of 37 mm (PRE) (Figure 5D) [28]. Additionally, mature fruits were observed between September and October 2014 (Figure 5A–D).

The foliage of the trees remained stable between November 2014 and January 2015. In February 2015, both juveniles and adults showed visibly increased foliage filling the canopy, which was more remarkable in the *angical* composed of juvenile individuals by the end of the month (T = 25 °C, RH = 69%, PRE = 239 mm [28]). In March 2015, the initial development of a small number of green fruits was observed. However, this phenophase is difficult to detect due to the height of the trees and the similar color of the pods and leaves. In August 2015, intense leaf loss was observed throughout the entire region where this deciduous forest occurs within the fragment (Figure 5E).

### 3.3. Genetic Characterization of AcolPM

#### 3.3.1. Analysis of PCoA and UPGMA

**Figure 6:**
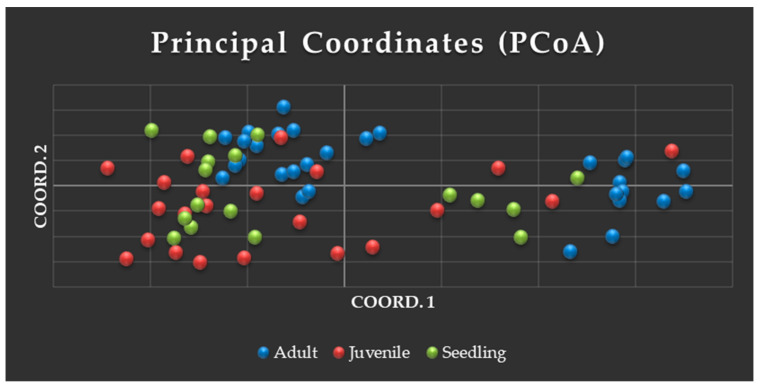

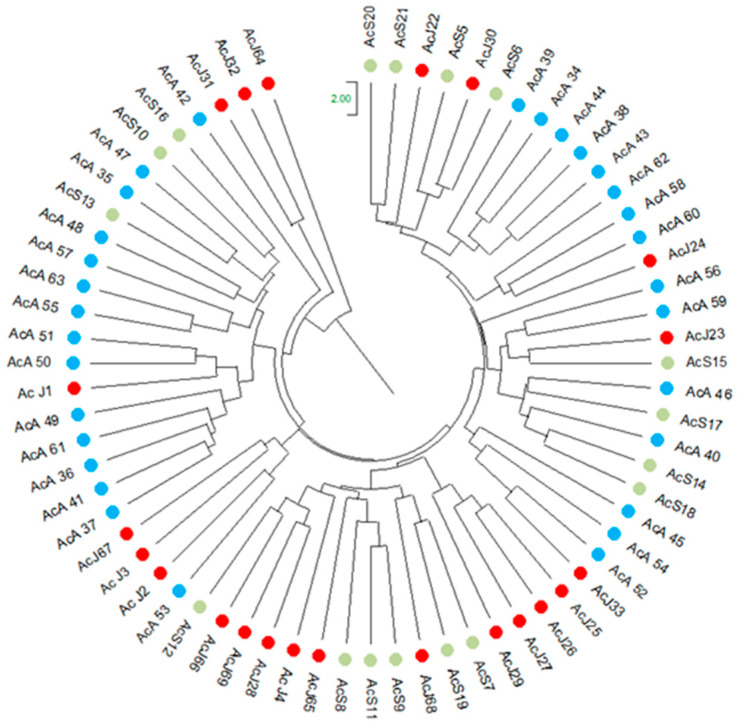
Principal Coordinate Analysis (PCoA) (**above**) and UPGMA dendrogram (**below**) showing the genetic relationships among adult (blue), juvenile (red), and seedling (green) individuals of *A. colubrina* (AcolPM) using 14 SSR markers.

In the Principal Coordinate Analysis (PCoA) (Figure 6), we observed two groupings of adult trees. Next to the first grouping on the left were observed most of the juvenile individuals and seedlings. The majority of individuals from the juvenile and seedling generations formed a group, regardless of their location within the fragment. Twelve seedlings clustered near the adult plants of the first group, as observed in the fragment, and five of them clustered near the second group of adults, although these were more geographically distant (Figure 6).

In the subgroups of the dendrogram, different clusters can be observed, formed by juvenile (J) and seedling (S) individuals (J22, J30, S5, S6, S20, S21; J4, J28, J65, J68, J69, J66, S7, S8, S9, S11, S12, S19), adults (A) and juveniles (A50, A51, J1; A45, A52, A54, J33), adults and seedlings (A35, A47, A48, A53, A55, A57, S10, S13, S16; A40, A46, S17, S14, S18), and exclusively adult individuals (36, 37, 41, 49, 61; 34, 38, 43, 44; 58, 60, 62). Only one small cluster was formed by individuals from all three generations (56, 59, J23, S15). The adult (42) and three juveniles (31, 32, 64) appeared separated from the other individuals (Figure 6). These graphs thus demonstrate different genetic associations among individuals of the three generations.

#### 3.3.2. Genetic Diversity Among Generations of *A. colubrina*

All 14 microsatellite loci analyzed were polymorphic (Tables S4 and S5). The loci Acol 10, Acol 13, and Acol 19 amplified the most alleles across the three generations, while Acol 5, Acol 11, and Acol 14 amplified the fewest alleles. For the Acol 5 locus, only one fixed allele was observed in the juvenile population and seedlings, with a second allele appearing only in individual 64 of the juvenile population.

We observed different total numbers of alleles in the *A. colubrina* generations, with 96, 89, and 82 alleles found in adult, juvenile, and seedling individuals, respectively (Table S5). There were 14, 7, and 5 exclusive alleles in adults, juveniles, and seedlings individuals, respectively, while 8 and 12 alleles were exclusive to juveniles + seedlings and adults + juveniles, respectively (Table S6).

The adult trees had the highest average number of alleles per locus (Na) (6.9) compared to the other generations (juveniles = 6.4, seedlings = 5.9). The average effective number of alleles per locus (Ne) was similar and lower (mean = 4.10) than the average number of alleles per locus (mean = 6.36) across the three generations, indicating that many alleles are of low frequency (Table 1, Table S6). The He (mean = 0.623) and Ho (mean = 0.538) were statistically similar between generations (Table 1). In the three generations, the mean heterozygosity observed was lower than the mean heterozygosity expected, and the average fixation index (mean = 0.125) was positive, indicating inbreeding.

#### 3.3.3. Inbreeding, Genetic Structure, and Genetic Distances

The inbreeding values (Fis inbreeding within subpopulations, Fit overall inbreeding) among plants within populations were positive and significantly different from zero (Table 2). This result suggests deviations from the proportions expected under Hardy–Weinberg equilibrium due to an excess of homozygotes, probably caused by the isolation of this population, increased mating between relatives, the reproduction system, and the barochorous seed dispersal of the species. Positive values of Fis (0.16) indicated inbreeding within individuals of the generations, suggesting greater homozygosity than would be expected randomly. This can occur due to mating between relatives and the reproduction system. The Fit value (0.194) indicated a reduction in observed heterozygosity compared to that expected in the total population, further suggesting the presence of inbreeding. The Fst values (0.04) showed low genetic divergence and a high historical gene flow rate (Nm = 6.7) among generations, indicating that the generations had low structuring and were not isolated in the past (Table 2).

The Analysis of Molecular Variance (AMOVA) showed that the greatest diversity was found within the sampled generations (94%), and the difference between generations was 6% (*p* < 0.001), values close to the Fst (Table 2).

The lowest genetic distance among generations was between juveniles and seedlings (4.8%), and the highest distance was between adults vs. juveniles (9.0%) and adults vs. seedlings (7.6%) (Table 3).

#### 3.3.4. Spatial Genetic Structure (SGS)

**Figure 7:**
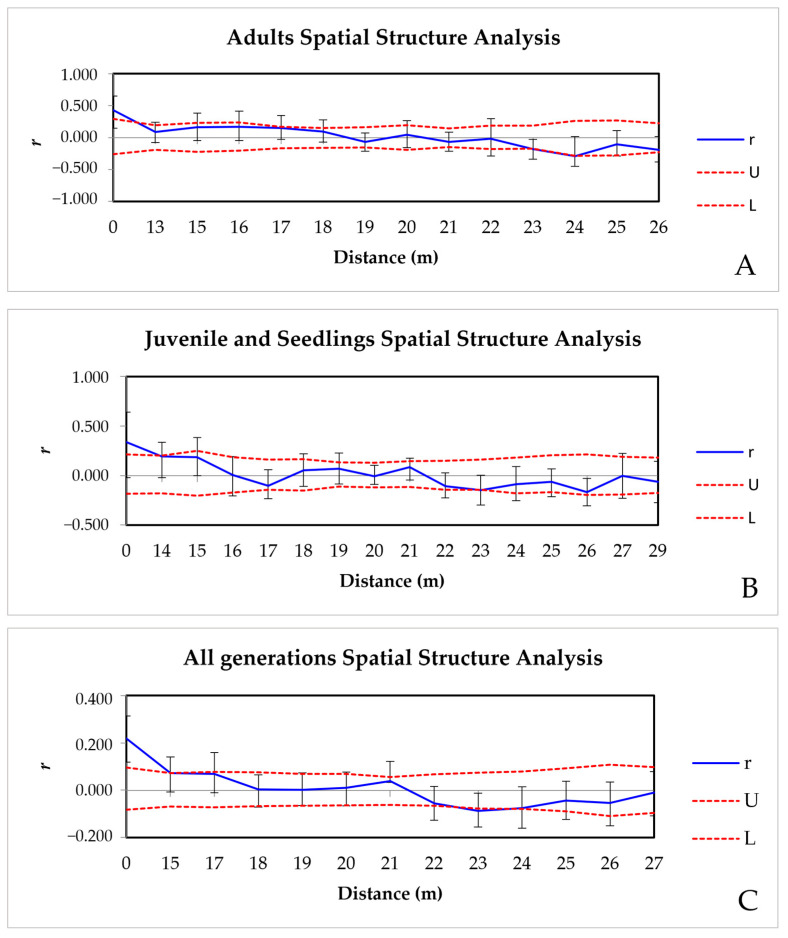
Spatial genetic structure (SGS) in the adult (**A**), juvenile + seedling (**B**), and all generations (**C**) from the AcolPM population, located in the M103 fragment, part of the Parque Municipal Morro de São Bento, Ribeirão Preto, SP, Brazil. The upper (U) and lower (L) red dashed lines represent the 95% confidence interval for *r* = 0, and the vertical bars indicate the 95% confidence interval for each *r*-value. Blue lines outside the confidence interval denote *r*-values significantly different from 0, indicating genetic structuring.

In the spatial genetic structure analyses, we observed a significant and positive correlation for adult and juvenile/seedling individuals up to 0–13 m (*r* = 0.428, *p* = 0.006) and 0–14 m (*r* = 0.193, *p* = 0.033) of distance, respectively (Figure 7A,B). In both analyses, there was a general decrease in *r* with distance, with a negative correlation at 23 m (*r*_adult_ = −0.178, *p* = 0.022; *r*_juvenile + seedling_ = −0.147, *p* = 0.024) (Figure 7A,B). When all individuals were analyzed as one population, a spatial genetic structure was observed up to 17 m (*r* = 0.069, *p* = 0.049) with a decrease at 23 m (*r* = −0.088, *p* = 0.010) and 24 m (*r* = −0.076, *p* = 0.032), demonstrating a diminished genetic relatedness between individuals at these distances (Figure 7C).

## 4. Discussion

In this research, we analyzed whether the resilience of 30 deciduous fragments, with high occurrence of the forest species *A. colubrina*, is due to their occurrence on land unsuitable for agriculture and urban expansion and how this contributes to their preservation and gene flow. Using the *A. colubrina* population from fragment M103 (AcolPM), which is located in a highly anthropogenically affected and geographically equidistant area, we studied how this urban matrix can affect its genetic diversity, inbreeding, and spatial genetic structure. Additionally, we studied its phenology and examined how its reproductive system and pollinators may allow its gene flow and resilience in the region of Ribeirão Preto, SP, Brazil.

Our results showed that the conservation of 17 from 30 fragments containing *A. colubrina* species in the region is associated with their specific occurrence on slopes greater than 12%, unsuitable for mechanized agriculture and urban expansion [39], which would collaterally imply their preservation. Therefore, the occurrence of this species can be considered an indicator of landscape transformations in the region. The connectivity within the pollination network, based on the documented maximum flight ranges of *A. colubrina* pollinators *A. mellifera* (6 km) and *T. spinipes* (8 km), demonstrated that the 8 km network established by the native pollinator encompassed all fragments. The *A. colubrina* AcolPM population from fragment M103 emerged as a prominent node, serving as a central hub of the shortest routes that link north to south fragments (as evidenced in the betweenness centrality analysis), which may indicate a significant influence on regional gene flow across the network (Figure 1B,C). Additionally, the connectivity analysis for 6 and 8 km highlighted the importance of the DJBOT fragment, along with D54 and D45 at 6 km and C121 and D17 at 8 km, further reflecting their roles within the pollination network (Figure 1B,C). However, these networks can potentially be more cohesive by integrating other types of forest formations, with approximately 70 fragments (not mapped here) [18] in the region that can also serve as sources of nectar and pollen for these pollinators.

Previous studies using ISSR molecular markers by our group in the AcolPM population and two others in the region (AcolUSP, and a smaller *angical* AcolBP) found similar diversity levels (~h = 0.30); nevertheless, AcolPM individuals formed a distinct cluster and exhibited greater genetic distances (~5%) than the other two populations. AcolBP and AcolUSP were also analyzed with the same SSR, which identified intermediate and similar diversity indices (~He = 0.56) and inbreeding (~F = 0.27). Extending these findings, in this manuscript, we observed significant inbreeding and spatial structure in the first meters of the AcolPM population. We also observed intermediate genetic diversity and low structuring among generations (Fst = 0.04) and different genetic associations among individuals of the three generations. Our results confirm our hypotheses and increase the knowledge about the species.

Genetic diversity is higher in tree species and populations with outcrossing breeding systems, seed dispersal by wind or animals, and wide geographic ranges compared to species with other combinations of features [41]. With barochorial seed dispersal, *A. colubrina* is a mixed-mating reproductive species (tm = 0.619, [9]) pollinated by Apidae bees, including *A. mellifera* and the native *T. spinipes* [10], which can travel kilometers in search of nectar and pollen. In this study, we observed that the average expected heterozygosity of the samples was intermediate (0.62) among AcolPM generations. This value was moderately higher than those found in other *angicais* in the region (AcolUSP = 0.57 [20]), possibly due to the spatial distribution of the fragments. For instance, AcolUSP is located within a fragment with low betweenness centrality in the overall fragment network based on native pollinator ranges (Figure 1B,C). The average He values across generations observed in *A. colubrina* in this study were also higher than those recorded in adults and seedlings of the AcolBP population (0.51 and 0.42, respectively [20]) yet lower than those from Argentinean samples (He = 0.66 [42]). These comparisons suggest that the observed He values are characteristic of the species. Additionally, among others, the He values were similar to those observed in other forest species, such as *Cariniana estrellensis* (He*_SII_* = 0.618, [43]) and *Aspidosperma polyneuron* (He*_SI_* = 0.65, [43]), but lower than those reported for *Metrodorea nigra* (He = 0.8, [44]), all located and studied in the Ribeirão Preto region, Brazil.

Inbreeding was observed in AcolPM population generations. These values may be influenced by the barochoric seed dispersal system, making this a mixed-mating reproductive species with a self-fertilization rate of 38%; the typical aggregation of the trees (*angicais*); the establishment of seedlings from spatially clustered shared parents; along with the population’s isolation. All these situations increase mating between related individuals [9,45] and can promote the spatial genetic structure (SGS). Our analyses of the SGS show that neighboring individuals of *A. colubrina*, including adults and juveniles/seedlings, exhibit higher genetic relatedness within the first distance classes (0–13 m and 0–14 m when analyzed separately, respectively, and 0–17 m when the entire population is analyzed), consistent with observations in Argentinean *A. colubrina* full siblings located spatially close to each other (from 1 to 15 m) [45]. The SGS observed at 17 m captures the interaction among all generations, consolidating the genetic pattern at a broader scale. This reflects how limited seed dispersal and the aggregation of parent trees influence genetic connectivity among individuals. The clustering of seedlings near parent trees observed in AcolPM aligns with this dispersal pattern, highlighting the biological significance of SGS at short distances. Previously, Feres et al. in 2021 [9] detected 15% inbreeding in the AcolBP population due to mating among relatives. They also observed that most crossings occurred locally, with moderate pollen dispersal (30%) from outside the fragment boundaries, indicating no reproductive isolation. Our results, in addition to genetic diversity, demonstrate that the matrix surrounding the fragments does not directly affect the levels of He and inbreeding, as also observed by Moraes-Filho et al. in 2025 [46]. This information can guide the definition of distances between mother trees used as a seed source for reforestation.

The duration, intensity, and synchrony of phenophases significantly impact the resources available to consumer organisms (pollinators, dispersers, and predators) and the regeneration of populations [47,48,49] as well as their genetic diversity, inbreeding, and spatial structure [46]. In tropical forests, budding synchrony is influenced by water and light availability [40,50,51]. Our observations of *A. colubrina* flowering (October) and fruit maturation (September–October) indicate that these events occur at the beginning of the rainy and hottest season. This is consistent with previous information for the species in São Paulo [8]. During the dry season, leaflessness was more prolonged in *A. colubrina* juvenile individuals, as the rate and degree of rehydration varied strongly with the capacity of water stored in tree trunks and its availability in the subsoil [40]. Generally, our findings align with those previously reported [51,52] for South American dry forests. In Brazil, *A. colubrina* phenology is variable, with flowering and fruit maturation differing by geographical location. For instance, the species flowers from July to November in Mato Grosso do Sul, in September in Piauí, and from September to November or early December in São Paulo. The fruits mature in October in Mato Grosso, in June in Piauí, and from April to October in São Paulo, as detailed by Carvalho in 1994 [8]. Although we only collected data over the course of one year, these observations are crucial pieces of information for understanding future climate change impacts and the resulting changes in plant species [53]. Additionally, local phenological characterization of *A. colubrina* is essential for seed collection aiming at conservation and reforestation.

Fragmentation and urbanization reduce plant species richness and pollinator availability, homogenize environments, promote non-native species invasion, and alter phenological events and plant growth [54]. However, species show adaptation to these adversities through phenotypic plasticity and evolved traits that enhance resilience [55]. Also, mating system plasticity (such as mixed-mating systems) allows plants to overcome reproductive challenges by adapting their phenology to flower earlier, using more self-pollen for quicker seed production [55], and by increasing selfing [12] or outcrossing [56] to cope with pollinator limitations or excess visitation. These traits, along with typical aggregation and phenological flexibility, enable *A. colubrina* to withstand and recover from natural and anthropogenic stresses, ensuring its survival.

The fragments of the deciduous and semi-deciduous Atlantic Forest are of great genetic and ecological value because they contain the last representative species of the native local flora [17,18,19,20,43,44,57,58,59]. Recently, the “Parque Municipal Morro de São Bento” in Ribeirão Preto was designated as an Environmental Protection Area (APA) by RP municipality decree No. 51 (14 March 2024). Therefore, its conservation and the protection of its species are of great significance to the region. In addition, preserving the populations of dominant forest species in the surviving forest fragments could be part of the regional government’s “Ribeirão −3 degrees” initiative [60] and help mitigate heat waves [61] and future temperature increases. Our findings support the conservation of this *A. colubrina* urban stand, along with other species in the fragment, and its use for future forest restoration of degraded areas. *A. colubrina* is a strategic species in this high-temperature region, helping to mitigate heatwaves and temperature increases, cooling with its shade (Annex S1 and Figure S1), and offering refuge and food for local insects and birds. We observed that the *angicais* help to reduce the soil temperature by 20 °C and air temperature by 7 °C when compared to neighboring urban environments while still providing a sub-canopy area with comfortable visibility to the human eye, as observed in the field visits and measured with the radiance and temperature quick survey (Annex S1 and Figure S1). This characteristic is possibly linked to the small leaf dimensions and populational distribution patterns, and it supports Acol planting in urban parks to reduce heat island effects.

We observed that the *angicais* help to reduce 20 °C (soil temperature) and 7 °C (air temperature at 1.70 m) when compared to neighboring urban environments, while still providing a sub-canopy area with comfortable visibility to the human eye, as observed in the field visits and measured with the radiance and temperature quick survey (Annex S1 and Figure S1).

In general, an enhanced understanding of the phenology, genetic diversity, inbreeding, and spatial distribution characteristics of dominant forest species, such as the resilient *A. colubrina*, a hypothetical prominent node for gene flow among fragments, along with other biological traits like reproductive mode and seed dispersal patterns, in the highly fragmented region of Ribeirão Preto, SP, Brazil, is crucial. This knowledge contributes significantly to both the conservation of isolated populations and to guiding seed collection for the restoration of degraded areas.

## 5. Conclusions

The connectivity flow diagrams among the fragments, using the documented maximum flight ranges of the *A. colubrina* pollinators *A. mellifera* and *T. spinipes*, demonstrated that the 8 km network of the native pollinators encompassed all fragments, with M103 (AcolPM) assuming a critical node for hypothetical gene flow among them. Additionally, it was observed that most fragment analyses are located on slopes greater than 12%, which comprise terrains unsuitable for mechanized sugarcane plantations according to state legislation [37]. Thus, the resilience of *A. colubrina* in these fragments makes it an indicator species of territorial transformation and segregation in Ribeirão Preto, SP, Brazil. Future studies could verify gene flow between extreme node populations to examine their genetic connections. Furthermore, assessment and conservation of pollinators over time, aiming to determine their resilience in a future of rising temperatures, may be of great importance for the survival of the species that depend on them.

When compared to previous studies of two populations (AcolUSP and AcolBP) inserted in urban agricultural and forest matrices, both with similar inbreeding levels (~F_mean_ = 0.27) versus AcolPM (F_mean_ = 0.125), it was concluded that the urban matrix surrounding the AcolPM population did not influence its inbreeding levels. This inbreeding is likely intensified by the natural mixed-mating system, limited seed dispersal (barochory), and pollinator behavior favoring geitonogamy, leading to the formation of clusters and favoring the spatial genetic structure (SGS). Conversely, pollen gene flow, mediated by bees such as *A. mellifera* and *T. spinipes*, acts as a population homogenizer, facilitating allele exchange between individuals and generations. This indicates that current pressures have not altered the genetic composition of the analyzed generations and that external gene flow is facilitated by pollinators’ flight capacity of up to 8 km. Therefore, the mating system, pollinator behavior, flight capacity, and seed dispersal are critical in shaping spatial genetic structure and reproductive success in *A. colubrina*.

The AcolPM population possesses higher diversity than AcolUSP and AcolBP and will be a source for seed collection considering its phenological characteristics that may show differences over time. This population is preserved, and different species and Acol seedlings are observed growing under its sub-canopy (Figure S1C,D). However, previous genetic distance studies compared to AcolUSP and AcolBP showed that AcolPM individuals are part of one cluster, separate from the others [21]. Therefore, seed collections for reforestation should be conducted in different populations, considering the observed SGS (spatial genetic structure) of 17 m. Future research should investigate the genetic diversity of M103 in comparison with Acol populations located in the DJBOT fragment as well as in D54, D45, C121, and D17. These sites represent hypothetical critical betweenness centrality nodes within the pollinator network identified in this study. Such an analysis would provide valuable insights into the relationship between regional gene flow and pollinator dynamics while also assessing their potential for optimized seed collection strategies.

These findings support the conservation of this *A. colubrina* urban stand, along with other species in the fragment, and its use for future forest restoration of degraded areas. *A. colubrina* is a strategic species in this high-temperature region, helping to mitigate heatwaves and temperature increases, cooling with its shade, and offering refuge and food for local insects and birds.

## Figures and Tables

**Table 1:** Genetic diversity across *Anadenanthera colubrina* (Vell.) Brenan generations analyzed using 14 simple sequence repeat (SSR) markers. %P = polymorphism percentage, N = number of individuals. Mean and SE over loci for each population for Na = number of alleles, Ne = effective number of alleles, H_O_ = observed heterozygosity, He = expected heterozygosity, F = fixation index ([He − Ho]/He). NS (non-statistically significant).

Pop		%P	N	Na	Ne	Ho	He	F
Adults	Mean	93	29.93	6.857	4.271	0.530 ^NS^	0.619 ^NS^	0.125 ^NS^
	SE	-	0.071	1.217	0.745	0.073	0.079	0.062
Juveniles	Mean	100	21.93	6.357	4.152	0.521 ^NS^	0.635 ^NS^	0.145 ^NS^
	SE	-	0.071	1.020	0.734	0.062	0.065	0.071
Seedlings	Mean	93	17.00	5.857	3.888	0.563 ^NS^	0.616 ^NS^	0.102 ^NS^
	SE	-	0.000	0.937	0.580	0.093	0.076	0.100
Grand Mean	Mean	95	22.95	6.357	4.104	0.538	0.623	0.125
	SE	2.4	0.833	0.603	0.390	0.043	0.041	0.045

**Table 2:** The inbreeding values (Fis, Fit) and genetic structure (Fst) among individuals within generations. Nm = historical gene flow rate.

All Pops.	Fis (*f*)	Fit (*F*)	Fst (Ɵ)	Nm
Mean	0.164 **	0.194 **	0.036 **	6.7
SE	0.056	0.058	0.009	
99% CI	0.019	0.042	0.016	
	0.305	0.339	0.060	

Ref. [33], ** *p* < 0.01.

**Table 3:** Nei’s unbiased measure of genetic distance.

Adults	Juveniles	Seedlings	
0.000			Adults
0.090	0.000		Juveniles
0.076	0.048	0.000	Seedlings

## Data Availability

Data are unavailable due to privacy, see Supplementary Materials.

## Supplementary Materials

The following supporting information can be downloaded at: https://www.mdpi.com/article/10.3390/genes16040388/s1, Table S1. Fragments with occurrence of *A. colubrina* populations in Ribeirão Preto, SP, Brazil (Kotchekoff-Henriques, 2003 [18]); Table S2. Sampled individuals of *A. colubrina* from the AcolPM population, located in the M103 fragment, part of the Parque Municipal Morro de São Bento, Ribeirão Preto, SP, Brazil; Table S3. Universal Transversal Mercator (UTM) coordinates and diameter at breast height (DBH) of individuals selected for phenological monitoring of *A. colubrina* from the AcolPM population, situated within the M103 fragment of Parque Municipal Morro de São Bento, Ribeirão Preto, SP, Brazil.; Table S4. Genetic characterization of 14 SSR molecular markers in 69 individuals of *A. colubrina*. Na = no. of alleles, Ne = effective alleles, Ho = observed heterozygosity, He = expected heterozygosity, F = fixation index, Ta = annealing temperature (°C); Table S5. Number of alleles per locus per population; Table S6. Allele frequencies per population (14 loci, 69 samples, 3 populations [exclusive alleles: adult: blue; juvenile: red; seedlings: green; adult + juvenile: brown; juvenile + seedlings: violet]); Annex S1. Quick micrometeorological survey during a heatwave in the understory of *A. colubrina* from the AcolPM population situated within the M103 fragment of Parque Municipal Morro de São Bento, Ribeirão Preto, SP, Brazil; Figure S1. A quick survey of micrometeorological parameters in the understory of *A. colubrina* trees from the AcolPM population situated in the Parque Municipal Morro de São Bento, Ribeirão Preto, SP, Brazil (A–D) and at two additional urban sites: one in front of a museum near the park (E) and another on a street in downtown Ribeirão Preto, SP, Brazil (F). In panel E, the background shows the edge of the municipal park. All measurements were conducted under full sunlight, with averages calculated from three readings. Panel G presents PAR, temperature, and relative humidity measurements carried out at ground level (examples in A–B). Panel H shows air temperature and relative humidity measured at four sites: AcolPM, museum near the park, city center, and São Paulo Universisty (USP) campus.

## Abbreviations

The following abbreviations are used in this manuscript:

AcolPM	*Anadenanthera colubrina* population from Parque Municipal Morro de São Bento (same as AcolBM in [21]).
AcolUSP	*A. colubrina* population from São Paulo University (USP), Ribeirão Preto Campus, Ribeirão Preto, SP, Brazil
AcolBP	*A. colubrina* population from Bomfim Paulista, a district of Ribeirão Preto, SP, Brazil
AMOVA	Analysis of Molecular Variance
PCoA	Principal Coordinate Analysis
SGS	Spatial genetic structure
SSR	Simple sequence repeats
