# Supplementary material for: Phenology and Spatial Genetic Structure of Anadenanthera colubrina (Vell.), a Resilient Species Amid Territorial Transformation in an Urban Deciduous Forest of Southeastern Brazil
Source: Genes (Basel). 2025 Mar 28;16(4):388. doi: 10.3390/genes16040388 (PMC12026557; doi:10.3390/genes16040388)
Supplement: Supplementary file 1 [file genes-16-00388-s001.zip › genes-3531198-supplementary.pdf]

genes

Phenology and Spatial Genetic Structure of *Anadenanthera colubrina* (Vell.), a Resilient Species amid Territorial Transformation in an Urban Deciduous Forest of Southeastern Brazil

Ana Lilia Alzate-Marin[1,2*], Paulo Augusto Bomfim Rodrigues[1], Fabio Alberto Alzate-Martinez[3], Gabriel Pinheiro Machado[1,4], Carlos Alberto Martinez[4], Fernando Bonifácio-Anacleto[1,2*]

[1] Plant Genetics Laboratory, Department of Genetics, Faculty of Medicine of Ribeirão Preto (FMRP-USP/RP), University of São Paulo, Ribeirão Preto, 14049-900, SP, Brazil; PABR: paulobomfim21@hotmail.com; GPM: gabrielpm_4@hotmail.com
[2] Graduate Program, Department of Genetics, Faculty of Medicine of Ribeirão Preto (FMRP-USP/RP), University of São Paulo, Av. Bandeirantes 3900, Ribeirão Preto 14049-900, SP, Brazil
[3] Nawi Spatial Design and Research, Ribeirão Preto 14040-160, SP, Brazil; e-mail: nawistudio.contact@gmail.com
[4] Department of Biology, Ribeirão Preto School of Philosophy, Science and Literature (FFCLRP), University of São Paulo, Av Bandeirantes 3900, Ribeirão Preto 14040-901, SP, Brazil. CAM: carlosamh@ffclrp.usp.br
* Correspondence: ALAM: anaalzate@fmrp.usp.br, FBA: bonifacioanacleto@usp.br

Academic Editor: Ana Isabel Honfi, Eric Javier Martínez and Anna Reutemann

Received: 28 February 2025
Revised: 25 March 2025
Accepted: 26 March 2025
Published: 28 March 2025

Copyright: © 2025 by the authors. Submitted for possible open access publication under the terms and conditions of the Creative Commons Attribution (CC BY) license (https://creativecommons.org/licenses/by/4.0/).

SUPPLEMENTARY DATA

Table S1. Fragments with Occurrence of *Anadenanthera colubrina* Populations in Ribeirão Preto, SP, Brazil (Kotchekoff-Henriques, 2003 [18])

	Fragment	Description	Soil type	Size (h)	Altitude (m)	X	Y	Slope (%)	Altitude (m) DEM ASTGTMV0031
1	D9	Riparian forest Rribeirão da Onça	Hydromorphic , LRa + LRd-1	247	528	192242	7639028	8	526
2	D12	Riparian forest stream Labareda	TT-2 + LRr / LV-2 / Hi1 + Hi-2 + Hi-3	29	551	194622	7638036	6	542
3	D17	–	Li -1 + TE - LRe + LRd-1	48	629	200998	7640708	12	627
4	D23	Boa Esperança farm	Lithosol – Li-1 + TE	16	–	205449	7644103	12	675
5	D24	Near the Bonfim Paulista District	Li-1 + TE	12	686	206393	7646080	12	610
6	D25	Near the Bonfim Paulista District	Lithosol (Li-1 + TE), Red Latosol (LRe + LRd-1)	17	688	207189	7642858	24	687
7	D26	Humaitá	LRe + LRd-1	13	730	209060	7642355	11	717
8	D34	Boa Esperança farm	Red Latosol –LRd-1	4	674	205801	7645315	13	653
9	D42	Small farm Rialto	Lithosol -TE + Li-1, Red Latosol - LRe + LRd-1	28	749	202554	7645876	12	743
10	D45	Limeirinha	Lithosol – Li-1, Red Latosol – LRe + LRa + LRd-1	4	656	211003	7647196	19	662
11	D48	Resfriado	Red latosol – LRe + LRd-1	3	742	212854	7645708	6	710
12	D50	Near the border with Cravinhos	Li-1 / TE	8	675	215616	7646447	8	659
13	D53	Small farm Retiro do Ipê	Red latosol – LRe + LRd-1	28	631	204580	7648394	15	611
14	D54	Invernada	Red latosol – LRe + LRd-1	4	638	205391	7648642	34	634
15	D57	–	LRe -Lithosol	4	651	212999	7648452	18	628
16	D58	–	LRe / Li-1	8	643	213716	7649399	27	636
17	D86	Curupira (Park - Reforestation)	Basalt outcrop	3	579	210028	7653517	9	576
18	D124	–	LE-2 - Hi-1	4	610	220067	7660094	11	594
19	D125	Piripau Hill	Li-1 / LE-2	17	607	222609	7660866	20	606
20	D139	–	LRa + LRd-1 / Hi-1	5	595	212573	7664658	5	556
21	D141	Vegetation fragments near the Pardo River	LRa + LRd-1	6	534	203298	7667815	9	509
22	D145	–	Hi-1	8	545	210118	7668060	5	533
23	M11	Tamboril farm	Red latosol – LRe + LRa + LRd-1	162	543	192932	7639190	10	548
24	M103	Municipal Park "Morro de São Bento"	Lithosol, Red Latosol on the slope	18	560	209088	7656149	18	579
25	C120	Ribeirão Verde Condominium	Dark Red Latosol – LE-2, Hydromorphic - Hi-1	9	542	214302	7660485	10	538
26	C121	–	LE-2	5	537	214575	7661712	5	541
27	USP-1	Near the USP/RP reforestation area	–	–	592	202630	7657269	23	596
28	USP-2	Near to the Bandeirantes entrance USP/RP	–	–	570	203235	7656209	10	584
29	AC	Café Avenue next to the USP roundabout	–	–	554	204797	7656126	5	558
30	DJBOT	Jardim Botânico Neighborhood	–	10	603	210084	7651662	17	597

USP=São Paulo University, Ribeirão Preto Campus
*USP-1, USP-2 and AC were added to original list [18]

Table S2. Sampled individuals of *Anadenanthera colubrina* from the AcolPM population, located in the M103 fragment, part of the Parque Municipal Morro de São Bento, Ribeirão Preto, SP, Brazil.

	**Individual**	**Generation**	**UTM (23K)**		**DBH**
			X	Y	**(m)**
1	Acol 34	Adult	209121	7656091	2.03
2	Acol 35	Adult	209118	7656036	3.38
3	Acol 36	Adult	209092	7655969	1.52
4	Acol 37	Adult	209086	7655972	2.59
5	Acol 38	Adult	209119	7655974	1.62
6	Acol 39	Adult	209119	7655992	1.67
7	Acol 40	Adult	209140	7656008	2.64
8	Acol 41	Adult	209127	7656027	1.35
9	Acol 42	Adult	209133	7656045	0.92
10	Acol 43	Adult	209137	7656066	0.91
11	Acol 44	Adult	209176	7656060	2.66
12	Acol 45	Adult	209189	7656060	1.90
13	Acol 46	Adult	209195	7656048	2.26
14	Acol 47	Adult	209194	7656069	3.16
15	Acol 48	Adult	209208	7656253	4.51
16	Acol 49	Adult	209218	7656116	1.42
17	Acol 50	Adult	209209	7656124	1.47
18	Acol 51	Adult	209226	7656239	1.29
19	Acol 52	Adult	209213	7656251	0.99
20	Acol 53	Adult	209217	7656244	1.10
21	Acol 54	Adult	209223	7656247	1.64
22	Acol 55	Adult	209230	7656254	1.90
23	Acol 56	Adult	209200	7656210	2.13
24	Acol 57	Adult	209170	7656253	2.94
25	Acol 58	Adult	209170	7656184	1.90
26	Acol 59	Adult	209189	7656167	1.32
27	Acol 60	Adult	209172	7656166	1.52
28	Acol 61	Adult	209187	7656180	2.36
29	Acol 62	Adult	209194	7656163	2.00
30	Acol 63	Adult	209160	7656147	2.53
	Mean				**1.99**

1	Acol1	Juvenile	209204	7656157	0.27
2	Acol 2	Juvenile	209206	7656166	1.34
3	Acol 3	Juvenile	209223	7656246	1.40
4	Acol 4	Juvenile	209231	7656183	1.27
5	Acol 22	Juvenile	209239	7656161	1.06
6	Acol 23	Juvenile	209234	7656161	0.61
7	Acol 24	Juvenile	209230	7656160	0.81
8	Acol 25	Juvenile	209234	7656140	0.10
9	Acol 26	Juvenile	209225	7656138	0.87

	**Individual**	**Generation**	**UTM (23K)**		**DBH**
			X	Y	**(m)**
Cont...					
10	Acol 27	Juvenile	209217	7656131	0.66
11	Acol 28	Juvenile	209236	7656135	0.41
12	Acol 29	Juvenile	209222	7656134	0.46
13	Acol 30	Juvenile	209235	7656137	0.46
14	Acol 31	Juvenile	209227	7656123	0.36
15	Acol 32	Juvenile	209225	7656125	0.41
16	Acol 33	Juvenile	209225	7656120	0.54
17	Acol 64	Juvenile	209089	7655939	1.34
18	Acol 65	Juvenile	209080	7655948	0.82
19	Acol 66	Juvenile	209082	7655947	0.71
20	Acol 67	Juvenile	209085	7655939	1.42
21	Acol 68	Juvenile	209102	7655913	0.59
22	Acol 69	Juvenile	209102	7655913	0.79
	Mean				**0.73**

	**Individual**	**Generation**	**UTM (23K)**		**Height(m)/ Number leaflets**
			**X**	**Y**	
1	Acol 5	Seedling	209173	7656061	1.0 m
2	Acol 6	Seedling	209173	7656061	1.2 m
3	Acol 7	Seedling	209173	7656061	5 leaf
4	Acol 8	Seedling	209173	7656061	2 leaf
5	Acol 9	Seedling	209173	7656061	13 leaf
6	Acol 10	Seedling	209173	7656061	2 leaf
7	Acol 11	Seedling	209173	7656061	5 leaf
8	Acol 12	Seedling	209173	7656061	4 leaf
9	Acol 13	Seedling	209173	7656061	10 leaf
10	Acol 14	Seedling	209173	7656061	4 leaf
11	Acol 15	Seedling	209173	7656061	3 leaf
12	Acol 16	Seedling	209173	7656061	1.0 m
13	Acol 17	Seedling	209173	7656061	0.5 m
14	Acol 18	Seedling	209173	7656061	3 leaf
15	Acol 19	Seedling	209173	7656061	1 leaf
16	Acol 20	Seedling	209173	7656061	1 leaf
17	Acol 21	Seedling	209173	7656061	4 leaf

Table S3. Universal Transversal Mercartor (UTM) coordinates and Diameter at breast height (DBH) of individuals selected for phenological monitoring of *Anadenanthera colubrina* from the AcolPM population, situated within the M103 fragment of Parque Municipal Morro de São Bento, Ribeirão Preto, SP, Brazil.

**Individual**	**UTM (23K)**		**DBH (m)**	**Individual**	**UTM (23K)**		**DBH (m)**
	**X**	**Y**			**X**	**Y**	
AcolJ-01	209263	7656178	0.49	AcolA-03	209271	7656316	2.36
AcolJ-02	209254	7656164	0.72	AcolA-25	209268	7656300	0.99
AcolJ-03	209263	7656175	0.75	AcolA-31	209220	7656237	1.33
AcolJ-04	209251	7656172	0.58	AcolA-32	209213	7656244	1.58
AcolJ-05	209279	7656187	0.50	AcolA-33	209232	7656246	2.35
AcolJ-06	209255	7656195	0.66	AcolA-34	209227	7656231	2.31
AcolJ-07	209261	7656176	0.38	AcolA-35	209226	7656214	1.17
AcolJ-08	209261	7656191	0.79	AcolA-36	209227	7656219	0.81
AcolJ-09	209272	7656182	0.28	AcolA-37	2092205	7656207	1.91
AcolJ-10	209262	7656198	0.45	AcolA-28	2092253	7656280	2.19
	Mean		**0.56**	Mean			**1.70**

Table S4. Genetic characterization of 14 SSR molecular markers in 69 individuals of *Anadenanthera colubrina*. Na=No. Alleles, Ne = Effective Alleles, Ho= Observed Heterozygosity, He= Expected Heterozygosity, F= Fixation Index. Ta= Annealing temperature (°C)

**Loci**	**Primer sequences (5 ′ – 3 ′)**[1]	**Repeat motif**[1]	**Size**[1] **(bp)**	**Na**	**Ne**	**Ho**	**He**	**F**	**Ta**[1] **(°C)**
Acol2	F TGGCTATCACTCTCGGACTTC<br>R CATAATGCAAACCGATGACC	(CTT)5	181	4.00	1.18	0.13	0.15	0.13	57
Acol5	F TGGACTTTTGCACTGGACAC<br>R CCCACCCACCTAGAACTGTC	(GA)2 A(GA)7	234	2.00	1.01	0.01	0.01	-0.01	58
Acol9	F CCAGGGTCCTCTCAGATTG<br>R ATGCTCCTCTCAACACACC	(CT)10 (AT)7	160	9.00	5.24	0.78	0.81	0.03	58
Acol10	F CGTATGTAGGTGGACTTAATATGC<br>R GCATGAAAGAAAGCTAATCACTC	(TG)3 C(GT)15<br>(GA)20	190	18.00	9.35	0.60	0.89	0.32	54
Acol11	F GGAAATGCAAGATCAGAGGCTC<br>R GTTTGGTTGATTGAGGATGTCCT	(CA)9	200	3.00	2.06	0.17	0.52	0.66	60
Acol12	F CCCCACATTTAGAACAGGAG<br>R CCACCTTGGATGATGTTTC	(TA)9	118	3.00	2.22	0.67	0.55	-0.21	58
Acol13	F CTTATTGCCTACTCCCTAACC<br>R GCATGGGGTCTTGATCC	(AG)15	139	15.00	10.63	0.88	0.91	0.02	58
Acol14	F GCGATACCCCCTTGATG<br>R GGCGAATGACCATCTTATC	(GA)11	183	3.00	1.95	0.58	0.49	-0.19	54
Acol15	F CATATGTCCAATCAGGTTAGAC<br>R CTCATTTGGTGACTGTAAGC	(GA)10	124	11.00	3.53	0.67	0.72	0.07	58
Acol16	F AAGGTCCAAGGGTTATGC<br>R GGACGTTCTCTTGTCATGC	(TC)11(CA)5<br>AA(CA)5	220	9.00	5.64	0.57	0.82	0.31	58
Acol17	F ACTCTCGACAACAAGTAATTCAAC<br>R CAACAGCCAATGACAATGATG	(AG)9	182	11.00	7.00	0.78	0.86	0.09	58
Acol18	F GTCAGGTATTCAATCTTCTACGT<br>R CACCATACCTCCACCTCATC	(TG)10	160	9.00	5.89	0.54	0.83	0.35	52
Acol19	F GACTGAAACCATTCTTTTATC<br>R TGTTGCGTATGTGGGTG	(CT)5 (CCCTCT)4<br>(CT)16 (AC)7	163	14.00	9.38	0.70	0.89	0.22	52
Acol20	F CAACCGCCACCATCAAAC<br>R AGTATACCAGTAGATCACAAGGATG	(GT)4 GGG(TG)6	136	5.00	2.87	0.41	0.65	0.37	56
Mean				8.29	4.85	0.54	0.65	0.16	
SE				1.37	0.87	0.07	0.08	0.06	

[1]Feres et al., 2012 [30]

Table S5. Number of alleles per locus per population

Locus	Pop 1 (30)	Pop2 (22)	Pop3 (17)
Acol2	2	4	2
Acol5	1	2	1
Acol9	8	8	7
**Acol10**	**14**	**13**	**12**
Acol11	3	3	2
Acol12	3	3	3
**Acol13**	**14**	**12**	**11**
Acol14	3	2	3
Acol15	7	7	6
Acol16	5	7	6
Acol17	10	7	9
Acol18	9	6	7
**Acol19**	**13**	**12**	**9**
Acol20	4	3	4
Total	**96**	**89**	**82**
Average	6.857	6.357	5.857
SE	1.217	1.019	0.936

Table S6. Allele Frequencies per Populations. (14 loci. 69 samples. 3 subpopulations [exclusive alleles Adult: blue; juvenile: red; seedlings:green. Adult+Juvenile:brown. Juvenile+seedlings:violet])

Locus	Allele/n	Adult	Juve	Seedl
		30	22	17
**Acol2a**	1	0.00	0.11	0.03
	3	0.02	0.05	0.00
	4	0.98	0.80	0.97
	5	0.00	0.05	0.00
**Acol5a**	1	1.00	0.98	1.00
	2	0.00	0.02	0.00
**Acol9a**	1	0.10	0.05	0.09
	2	0.20	0.25	0.15
	3	0.30	0.23	0.24
	4	0.27	0.18	0.32
	5	0.05	0.07	0.12
	6	0.00	0.09	0.00
	7	0.03	0.00	0.00
	8	0.02	0.02	0.03
	12	0.03	0.11	0.06
**Acol10a**	1	0.27	0.17	0.18
	2	0.07	0.07	0.35
	3	0.03	0.02	0.03
	4	0.05	0.05	0.00
	5	0.00	0.05	0.00
	6	0.00	0.07	0.03
	7	0.03	0.00	0.00
	8	0.02	0.12	0.03
	9	0.07	0.02	0.03
	10	0.07	0.00	0.03
	11	0.07	0.07	0.03
	12	0.05	0.19	0.12
	13	0.00	0.00	0.03
	14	0.02	0.00	0.00
	15	0.02	0.07	0.06
	18	0.20	0.02	0.09
	19	0.05	0.00	0.00
	23	0.00	0.07	0.00
**Acol11a**	1	0.53	0.71	0.71
	2	0.40	0.11	0.29
	3	0.07	0.18	0.00
**Acol12a**	1	0.68	0.64	0.44
	2	0.08	0.14	0.29
	3	0.23	0.23	0.27
**Acol13a**	1	0.02	0.00	0.06
	2	0.12	0.14	0.00
	3	0.03	0.02	0.00
	4	0.05	0.05	0.09
	5	0.03	0.14	0.15
	6	0.15	0.11	0.12

Locus	Allele/n	Adult	Juve	Seedl
		30	22	17
**Acol15a**	1	0.07	0.05	0.09
	2	0.00	0.00	0.09
	4	0.43	0.55	0.41
	6	0.00	0.00	0.03
	8	0.00	0.05	0.00
	10	0.03	0.00	0.00
	11	0.18	0.05	0.15
	12	0.17	0.25	0.24
	16	0.00	0.05	0.00
	17	0.08	0.00	0.00
	18	0.03	0.02	0.00
**Acol16a**	1	0.00	0.07	0.12
	2	0.00	0.07	0.15
	3	0.00	0.27	0.32
	4	0.00	0.11	0.15
	5	0.28	0.30	0.15
	6	0.42	0.14	0.12
	7	0.10	0.05	0.00
	8	0.18	0.00	0.00
	9	0.02	0.00	0.00
**Acol17a**	1	0.05	0.00	0.00
	2	0.15	0.11	0.21
	3	0.17	0.36	0.15
	4	0.25	0.05	0.24
	5	0.12	0.18	0.03
	6	0.10	0.05	0.09
	7	0.03	0.09	0.09
	8	0.07	0.16	0.09
	9	0.02	0.00	0.00
	10	0.05	0.00	0.06
	11	0.00	0.00	0.06
**Acol18a**	1	0.13	0.09	0.44
	2	0.17	0.34	0.24
	3	0.22	0.25	0.03
	4	0.03	0.05	0.03
	5	0.03	0.14	0.21
	6	0.25	0.14	0.03
	7	0.12	0.00	0.00
	10	0.02	0.00	0.03
	11	0.03	0.00	0.00
**Acol19a**	1	0.07	0.02	0.03
	2	0.02	0.00	0.00
	3	0.15	0.05	0.00
	4	0.15	0.02	0.00
	5	0.12	0.02	0.00

Table S6. Continuation...

Locus	Allele/n	Adult	Juve	Seedl	Locus	Allele/n	Adult	Juve	Seedl
		30	22	17			30	22	17
**Acol13a**	1	0.02	0.00	0.06	**Acol19a**	7	0.05	0.00	0.00
	2	0.12	0.14	0.00		8	0.05	0.02	0.06
	3	0.03	0.02	0.00		9	0.15	0.05	0.15
	4	0.05	0.05	0.09		10	0.03	0.23	0.21
	5	0.03	0.14	0.15		11	0.02	0.07	0.18
	6	0.15	0.11	0.12		12	0.17	0.25	0.21
	7	0.13	0.07	0.03		13	0.00	0.21	0.06
	8	0.17	0.14	0.24		14	0.02	0.05	0.03
	9	0.00	0.07	0.06		16	0.02	0.02	0.09
	10	0.02	0.05	0.03	**Acol20a**	1	0.59	0.30	0.44
	12	0.05	0.05	0.00		2	0.07	0.18	0.00
	13	0.08	0.00	0.00		3	0.19	0.52	0.41
	14	0.02	0.11	0.12		4	0.00	0.00	0.06
	15	0.05	0.00	0.03		5	0.16	0.00	0.09
	16	0.08	0.07	0.09					
**Acol14a**	1	0.68	0.59	0.68					
	2	0.10	0.00	0.06					
	3	0.22	0.41	0.27					

Annex S1. Quick micrometeorological survey during a heatwave in the understory of *Anadenanthera colubrina* from the AcolPM population, situated within the M103 fragment of Parque Municipal Morro de São Bento, Ribeirão Preto, SP, Brazil.

On February 15, 2025, during the heatwave that occurred in the State of São Paulo, we conducted a rapid survey to record the Photosynthetically Active Radiation (PAR, μmol/m²/s), relative humidity (%), and temperature (°C) under a sub-canopy of *Anadenanthera colubrina* trees at Morro de São Bento, Ribeirão Preto, SP, Brazil (AcolPM). We used a TESTO 835-H1 infrared laser temperature meter (Testo, Germany) for temperature and air humidity measurements, and a LI250-A quantum meter (LI-COR, Nebraska, USA) for PAR measurements (See Supplementary Figure S1). This area of the forest, open to the public with a trail, is where people gather for leisure and to rest in the shade of *Anadenanthera colubrina* and other tree species that grow beneath its canopy (See Figure S1, panel C, D).

We collected PAR, temperature and relative humidity data (3 repetitions) on the "paved path between the trees with sunlight," on the "forest floor in the understory with sunlight," and at two other points in the city (23K 208995.65mE 7655809.44mS, 23K 208067.07mE 7655864.26mS), located approximately 22 and 1,000 meters from the park border, respectively (Figure S1). The micrometeorological data were collected between ~11:30 and 14:30 hours. We also collected temperature and relative humidity data at 1.70 m in all locations (air measures). Additionally, we used temperature and relative humidity data collected with Delta-T Devices (UK) sensors installed at 2 meters above the ground from the climatic station (WS-GP2) at São Paulo University, Ribeirão Preto Campus (USP-RP) (23K 202620.78mE 7656469.08 mS), between 12.00 and 14:00 hours. The distance between AcolPM and a USP-RP climatic station is ~6.6 kilometers.

The survey revealed that the sub-canopy radiation and temperature measurements taken at ground level on the paved path were, on average, 800 μmol/m²/s and 20 °C lower than those taken at the two other points in the city. Additionally, the mean relative humidity in the sub-canopy was 17% higher than at the other two locations (Figure S1, panel G). Measurements of air temperature and relative humidity showed that the temperature was, on average, 7 °C lower and the relative humidity was 17% higher in the sub-canopy of *Anadenanthera colubrina* trees compared to those observed at the other two points in the city (Figure S1, panel H). Both measurements were similar between AcolPM and the climatic station of USP-RP, although the climatic station is located in an open field, the campus as a whole is situated within a large forest cover.

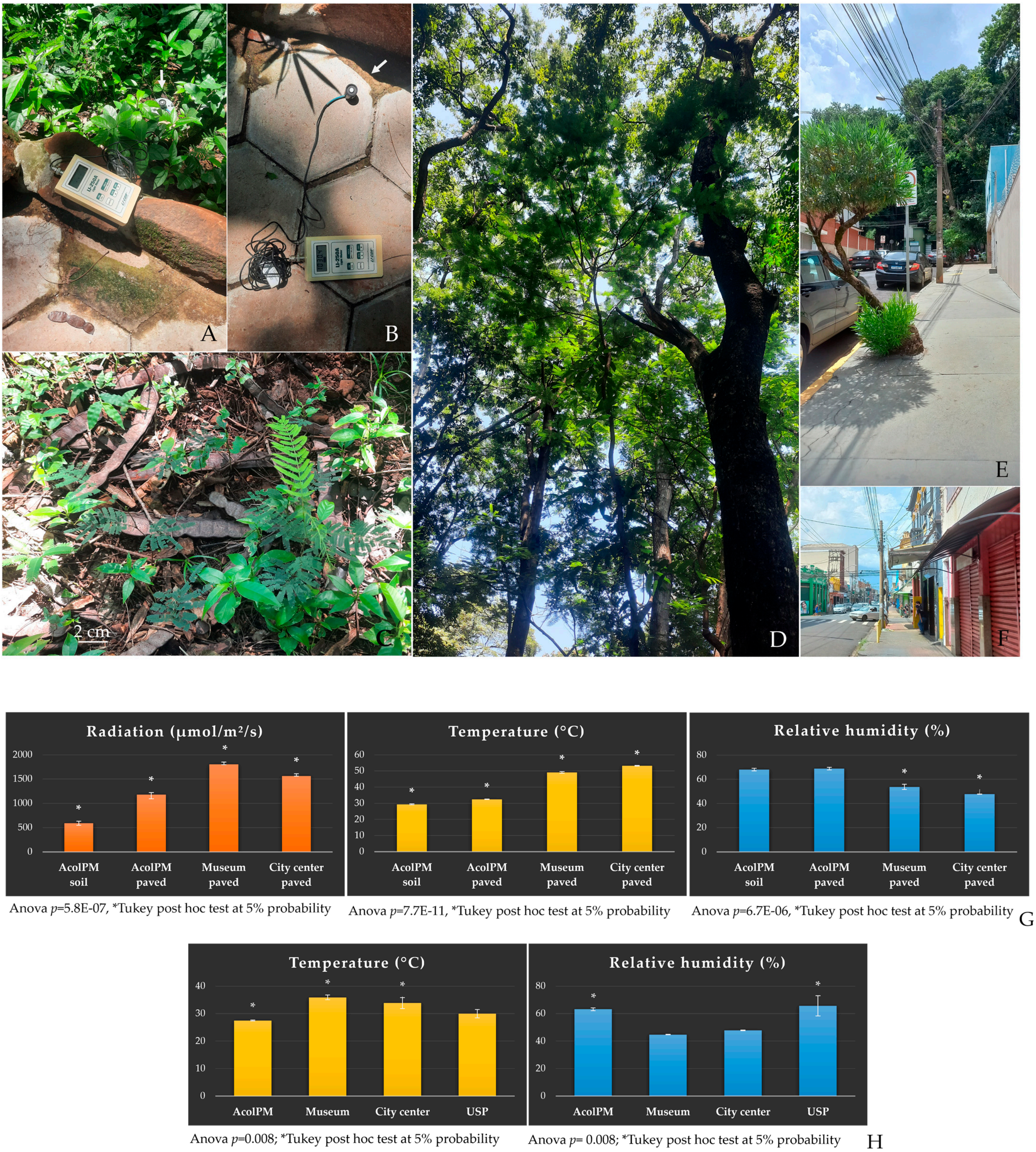

Figure S1. A quick survey of micrometeorological parameters in the understory of *Anadenanthera colubrina* trees from the AcolPM population, situated in the Parque Municipal Morro de São Bento, Ribeirão Preto, SP, Brazil (A–D), and at two additional urban sites: one in front of a museum near the park (E) and another on a street in downtown Ribeirão Preto, SP, Brazil (F). In panel E, the background shows the edge of the Municipal Park. All measurements were conducted under full sunlight, with averages calculated from three readings. Panel G presents PAR, temperature, and relative humidity measurements carried out at ground level (examples in A–B). Panel H shows air temperature and relative humidity measured at four sites: AcolPM, Museum near the park, City Center and São Paulo Universisty (USP) campus.
